# Editorial for the Special Issue “Advances in Viral Metagenomics”

**DOI:** 10.3390/microorganisms14071570

**Published:** 2026-07-17

**Authors:** Noely Evangelista Ferreira, Maria Cassia Mendes-Correa, Antonio Charlys da Costa

**Affiliations:** Laboratório de Investigação Médica—LIM 52, Instituto de Medicina Tropical, Faculdade de Medicina, University of São Paulo, São Paulo 05403-000, Brazil; noely.evangelista@hc.fm.usp.br (N.E.F.); maria.cassia@hc.fm.usp.br (M.C.M.-C.)

Viral metagenomics has fundamentally transformed how we investigate the virosphere. By bypassing cultivation and enabling direct sequencing of nucleic acids from environmental, animal, and human samples, this approach has revealed an extraordinary diversity of viruses, including many that were previously unknown or considered uncultivable [1]. The field has moved from proof-of-concept studies to a mature discipline that now informs public health surveillance, clinical diagnostics, evolutionary biology, and ecosystem ecology [2]. High-throughput sequencing platforms are increasingly accessible, bioinformatics pipelines are more sophisticated, and novel experimental strategies, ranging from virus-like particle enrichment to long-read sequencing, continue to expand the resolution with which viral communities can be characterized [3].

Nevertheless, important challenges remain. Distinguishing genuine novel viruses from sequencing artifacts, assembling fragmented genomes from complex metagenomes, predicting viral hosts with confidence, and establishing causal links between viral detection and disease are still non-trivial tasks. Against this backdrop, this Special Issue, “Advances in Viral Metagenomics” (freely available at https://www.mdpi.com/journal/microorganisms/special_issues/BQR7NN7Z90, accessed on 17 July 2026), was conceived to bring together original research and reviews that highlight methodological innovation, broaden our understanding of viral diversity across ecosystems, and explore the translational potential of metagenomic approaches [4,5,6,7,8,9,10,11,12].

This Special Issue features nine peer-reviewed papers that have collectively attracted substantial readership, reflecting the broad interest and relevance of the topic. The contributions span diverse geographic regions, host systems, and clinical and environmental contexts, addressing several important gaps in the literature [4,5,6,7,8,9,10,11,12].

Several studies in this issue focus on viral discovery in wildlife, with bats emerging as a recurring theme given their recognized role as reservoirs of viral diversity [1,2]. One study employed next-generation sequencing to characterize totivirus-like viruses in liver tissue from *Molossus molossus* bats in the Brazilian Amazon, identifying sequences belonging to two distinct phylogenetic clades and sharing less than 18% similarity with a previously described totivirus from bat guano [9,10]. This work suggests that the diversity of totiviruses in bats is likely far more extensive than previously recognized and highlights the importance of continued surveillance in under-sampled regions. Another study, analyzing bat fecal samples from multiple Russian cities, demonstrated the value of integrating advanced computational methods with experimental strategies by combining homology-based identification with targeted amplification to recover longer genomic fragments of novel picornavirus-like sequences. Together, these studies illustrate how viral metagenomics, applied across different continents and bat species, can reveal both shared patterns and region-specific viral lineages [11].

Methodological rigor is a second major theme. One of this issue’s most valuable contributions directly addressed how experimental protocols shape the conclusions drawn from virome studies. By comparing bulk metagenome approaches with two distinct virus-like particle enrichment protocols applied to human fecal samples, the authors systematically evaluated each method’s performance in terms of compositional representation, reproducibility, and taxonomic coverage. Their findings provide practical guidance for researchers designing virome studies and reinforce that no single protocol is universally superior; rather, the choice of method must align with the specific research question. This kind of critical methodological assessment is essential as the field matures and seeks to standardize practices across laboratories [4,6].

Clinical applications are equally well represented. One study applied metagenomic next-generation sequencing to cerebrospinal fluid from patients with central nervous system infections of unknown etiology in Brazil, reporting the first detection of human pegivirus genotype 2 in South American cerebrospinal fluid samples. While the direct pathogenic role of this virus in central nervous system disease remains uncertain, the finding opens new avenues for investigating the contribution of commensal or opportunistic viruses to neurological conditions. In the pediatric respiratory domain, another paper used pooled nasopharyngeal swabs from children who tested negative for SARS-CoV-2 during the Omicron wave, revealing that respiratory syncytial virus and enteroviruses were the most prevalent agents, alongside clinically significant detections of mumps virus, human metapneumovirus, influenza A, and multiple human herpesviruses. This work serves as a timely reminder that, while pandemic pathogens command global attention, endemic respiratory viruses continue to impose a substantial burden on child health, and that metagenomics offers an unbiased window into their circulation [7,8,12].

Looking ahead, several priorities emerge from the collective findings of this Special Issue. First, the field must continue to invest in standardization and benchmarking. As the comparative methodology paper demonstrates, seemingly minor differences in sample processing can profoundly affect observed virome composition. The development of reference materials, community-wide proficiency tests, and openly shared bioinformatics workflows will be critical to ensuring that results from different laboratories are comparable and reproducible.

Second, the integration of metagenomics with functional assays represents a necessary next step. Viral detection, however comprehensive, does not equate to viral activity or pathogenic potential. Combining metagenomic sequencing with transcriptomics, proteomics, or targeted serological surveys will help bridge the gap between sequence-based discovery and biological relevance. This is particularly important in clinical contexts, where the distinction between a passenger virus and a causative agent remains a central interpretive challenge.

Third, the expansion of metagenomic surveillance beyond well-studied populations and geographic regions is long overdue. The bat studies in this issue, from Brazil, Florida, and Russia, exemplify the value of geographic breadth, but vast areas of the planet, particularly in tropical and subtropical regions, remain under-sampled. Zoonotic spillover events do not respect national borders, and a truly global picture of viral diversity is essential for pandemic preparedness.

Finally, the computational challenges of viral metagenomics demand continued innovation. The identification of highly divergent viruses, accurate taxonomic assignment, host prediction, and assembly of complete or near-complete genomes from complex metagenomes all remain active areas of method development. Machine-learning approaches, hybrid assembly strategies, and improved reference databases will all play important roles in advancing the field.

The nine papers gathered in this Special Issue reflect the vitality and diversity of contemporary viral metagenomics research. They demonstrate that the field is no longer merely descriptive but is increasingly capable of generating actionable insights, whether they be in clinical diagnosis, environmental monitoring, or pandemic surveillance. The strong readership and engagement generated by these papers confirm that viral metagenomics has secured its place as a cornerstone of modern virology. We thank all the authors, reviewers, and editorial staff who contributed to this collection, and we hope that the work presented here will inspire further investigations into the hidden viral universe that surrounds us.
